# Development of a Multipurpose GATEWAY-Based Lentiviral Tetracycline-Regulated Conditional RNAi System (GLTR)

**DOI:** 10.1371/journal.pone.0097764

**Published:** 2014-05-19

**Authors:** Reinhard Sigl, Christian Ploner, Giridhar Shivalingaiah, Reinhard Kofler, Stephan Geley

**Affiliations:** 1 Division of Molecular Pathophysiology, Biocenter, Medical University of Innsbruck, Innsbruck, Austria; 2 Tyrolean Cancer Institute, Innsbruck, Austria; 3 Univ.-Clinics for Plastic, Reconstructive and Aesthetic Surgery, Medical University Innsbruck, Innsbruck, Austria; University of Georgia, United States of America

## Abstract

RNA interference (RNAi) has become an essential technology for functional gene analysis. Its success, however, depends on the effective expression of RNAi-inducing small double-stranded interfering RNA molecules (siRNAs) in target cells. In many cell types, RNAi can be achieved by transfection of chemically synthesised siRNAs, which results in transient knockdown of protein expression. Expression of double-stranded short hairpin RNA (shRNA) provides another means to induce RNAi in cells that are hard to transfect. To facilitate the generation of stable, conditional RNAi cell lines, we have developed novel one- and two-component vector **G**ATEWAY-compatible **l**entiviral **t**etracycline-regulated **R**NAi (GLTR) systems. The combination of a modified RNA-polymerase-III-dependent H1 RNA promoter (designated ‘THT’) for conditional shRNA expression with different lentiviral delivery vectors allows (1) the use of fluorescent proteins for colour-coded combinatorial RNAi or for monitoring RNAi induction (pGLTR-FP), (2) selection of transduced cells (pGLTR-S), and (3) the generation of conditional cell lines using a one vector system (pGLTR-X). All three systems were found to be suitable for the analysis of essential genes, such as CDC27, a component of the mitotic ubiquitin ligase APC/C, in cell lines and primary human cells.

## Introduction

RNA interference (RNAi) has advanced into an essential tool for functional gene analysis [1]-[3]. It exploits a conserved gene regulatory mechanism activated by double-stranded RNA (dsRNA) molecules that are processed into small interfering RNA (siRNA) molecules by the type III endoribonuclease DICER. Individual siRNA strands are then incorporated into the multisubunit RNA-induced silencing complex (RISC) to serve as guide RNAs for the identification, binding and subsequent RISC endonuclease-dependent cleavage of complementary target mRNAs, which leads to their rapid degradation and subsequent decline in protein levels (reviewed in [4], [5]).

The RNAi pathway can be activated by two means; delivery of synthetic siRNAs, which induces a transient knockdown of protein expression, or by expression of dsRNA precursor molecules that are processed by the cellular RNAi machinery into siRNAs, which results in longer lasting gene knockdown [6]. These dsRNA precursors are often expressed as short hairpin RNA (shRNA) molecules from RNA polymerase-III-dependent promoters. After their transcription, shRNA molecules are processed by the RNAse-III enzyme DICER to generate 19–21 bp long dsRNA molecules harbouring 2 nucleotide long 3′ extensions, which are characteristic of siRNAs [6]. Alternatively, the dsRNA precursors can be expressed within the context of micro-RNA (miRNA) molecules, expressed from RNA polymerase-II-dependent promoters. These dsRNA precursors are first processed by nuclear DROSHA, another member of the RNAse-III family, to release the pre-miRNA from the primary RNA transcript and then by DICER to generate siRNAs in the cytoplasm [7].

All three systems are widely used for RNAi experiments that include genome-wide loss-of-function screens in selected human cell lines and the establishment of transgenic model organisms for functional gene analysis. The success of an RNAi experiment crucially depends on the choice of the target sequence as well as the efficacy of siRNA expression, which has to be optimised for each cell line and adapted for experimental requirements. Thus, while for certain experiments in some cell lines transient transfection of synthetic siRNAs is the optimal strategy, expression of shRNAs might be more suitable in other circumstances and the best RNAi strategy has often to be determined experimentally. To overcome the limitations of transfection technologies, shRNAs are frequently expressed from viral vectors, including adeno-, retro- and lentiviral vectors, which also allow the generation of stable RNAi cell lines [8], [9]. When analysing essential genes, however, shRNA expression in stable cell lines has to be conditional.

Several different conditional RNAi systems have been developed over the past decade [10]–[14]. The most frequently used systems are based on the expression of shRNAs from conditional RNA polymerase-III-dependent promoters [15]. Because siRNAs can also be processed from miRNAs, a variety of cell type specific and conditional RNA polymerase-II-dependent promoter systems have been used for siRNA expression [13]. In addition to these often somewhat leaky systems, more tight expression systems, such as Cre-recombinase mediated deletion of a ‘floxed-stop’ cassette, have been successfully used in cells as well as in transgenic animals [12], [16]. The establishment of such conditional RNAi systems usually requires multiple transgene insertions with at least two vectors, subsequent selection and evaluation, which is time and resource consuming and precludes their use in non- or slowly proliferating primary cells. To overcome these limitations and to facilitate the rapid generation of diverse delivery vectors, we developed a novel lentiviral GATEWAY-cloning based vector system for tetracycline dependent conditional RNAi and evaluated it by targeting an essential gene required for progression through mitosis.

## Materials and Methods

### Reagents

All chemicals were obtained from Sigma (Vienna, Austria), enzymes from Promega (Mannheim, Germany) and oligonucleotides from MWG Biotech (Ebersberg, Germany) or Microsynth AG (Balgach, Switzerland), unless stated otherwise.

### Plasmid Construction

The THT promoter was constructed by first subcloning the H1-RNA gene promoter as a SmaI-HinDIII fragment of pSUPER (kindly provided by Reuven Agami, NKI, Amsterdam, Netherlands) into the respective sites of pUHD10-3 (kindly provided by Manfred Gossen, MDC, Berlin, Germany), followed by PCR amplification using primers 5′-CTGCAGGAATTCGAACGCTGACG-3′ and 5′-TATAGATCTCTATCACTGATAGGGACTTATAAGATTCCCAAATCCAAAG-3′ to introduce a TetR binding site (underlined) downstream of the TATA box, and subcloning into the episomal expression vector pEPU, a derivative of pCEP-Pu [17] lacking the CMV promoter. To create pENTR-THT, the THT promoter was excised from the episomal plasmid using BamHI and PvuII and blunt-end cloned into the NotI BamHI digested and filled-in pSHAG1 (kindly donated by Greg Hannon, CSHL, New York, NY, USA). After sequencing, a 1.3 kb BglII-HinDIII stuffer fragment was subcloned from pEF-YFP [18] into the BglII-HinDIII sites of pENTR-THT to generate pENTR-THT. pENTR-THT-III was generated by subcloning the THT promoter into pDONR-207 (Invitrogen, Vienna, Austria) after its BglII site in the gentamycin resistance gene was disrupted by site-directed mutagenesis. pENTR-H1 was constructed by subcloning the H1-promoter containing EcoRI-SalI fragment of pRETRO-SUPER (kindly provided by Reuven Agami) into the respective sites of pENTR-1A (Invitrogen, Vienna, Austria).

The lentiviral GATEWAY destination vector pHR-DEST-GFP ( = pGLTR-FP-GFP) was generated by inserting a DEST cassette into the blunt-ended EcoRI site of pHR-SIN-CSGW (kindly provided by Mary Collins, UCL, London, UK). Plasmid pHR-DEST-dtTOMATO ( = pGLTR-FP-RFP) was made by exchanging the GFP cassette in pHR-DEST-GFP with that for dtTOMATO (kindly provided by Roger Tsien, UCSD, San Diego, CA, USA). The selectable lentiviral construct pHR-DEST-PURO ( = pGLTR-S-PURO) was constructed by exchanging GFP with the puromycin N-acetyl transferase (pac) gene [19]. The single vector RNAi plasmid pHR-DEST-TetR-GFP ( = pGLTR-X-FP) was made by amplifying TetR-NLS from pEF-TetR-KRAB [20] in two PCRs using 5′-TATAGGATCCGCCACCATGGCTAGATTAGATAAAAGTAAAGTGATTAACA-3′ and 5′-CCACATCGCCGCAGGTCAGCAGGCTGCCGCGGCCTTCACCACCGCCGTCG-3′ in the first and 5′-TATAGGATCCCCCGGGCCCGGGTTTTCTTCCACATCGCCGCAG-3′ in the second reaction (which introduced a T2A sequence between TetR and eGFP). The PCR product was digested with BamHI and sublconed into pHR-SIN-CSGW upstream of eGFP. After sequencing, a DEST cassette was inserted as described above. For further modifications of pGLTR-X-FP, a KpnI-NdeI fragment containing the ‘NLS-T2A-eGFP’ region was subcloned into KpnI-NdeI digested pUC19, generating pUC19-NLS-T2A-eGFP. The pac gene fragment was PCR amplified from pGLTR-S-PURO with primers 5′ATATACCGGTCGCCACCATGGCCATGACCGAGTACAAG-3′ and 5′- ATATGCGGCCGCTTCAGGCACCGGGCTTGCGGG-3′ and digested with AgeI and NotI to replace the eGFP fragment. In a second step, the NLS-T2A-Puro containing KpnI-NdeI fragment from pUC19-NLS-T2A-Puro was subcloned back into pGLTR-X-FP, resulting in pGLTR-X-PURO. pENTR-THT-CDC27 was created by cloning 5′-end phosphorylated and annealed oligos 5′- GATCCCCGCCAGATCCTGACCAAACATTCAAGAGATGTTTGGTCAGGATCTGGCTTTTTGGAAA-3′ and 5′- AGCTTTTCCAAAAAgccagatcctgaccaaacatctcttgaatgtttggtcaggatctggcGGG-3′ into BglII-HinDIII digested, dephosphorylated pENTR-THT-I (the sense target sequence is underlined). After isolation of recombinant plasmids, the insert was amplified using primers 5′-CTGGAGGAATTCGAACGCTGACG-3′ and 5′-TGTAAAACGACGGCCAGT-3′, and DNA sequenced on an AB 5500 XL Solid Sequencer (Life Technologies, Vienna, Austria). The THT-shRNA expression cassette was subsequently transferred into GLTR vectors using standard LR recombinase reactions (Invitrogen). The retroviral expression vector pLIB-TetR-KRAB-IRES-BLAS was constructed by subcloning TetR-KRAB from pEF-TetR-KRAB [20] into pLIB-MCS2-IRES-BLAS using primers 5′-TATAAGATCTGGATCCACCATGGCTAGATTAGATAAAAGTAAAGTG-3′ and 5′-TATAGATATCTCAGGCACCGGGCTTG-3′. All described plasmids are deposited at the plasmid distribution platform Addgene (www. addgene.com).

### Cell Lines and Primary Cells

U2OS (ATTC:HTB-96), HEK293T, HEK293A (Invitrogen, Vienna, Austria) and the PHOENIX amphotropic retroviral packaging cell line [21] (kindly provided by G.P. Nolan, Stanford, CA, USA) were grown in DMEM supplemented with 10% FCS, 100 µg/ml streptomycin and 100U/ml penicillin in saturated humidity at 37°C, 5% CO2. Leukemic PREB697/EU3 cells (ACC 42, DSMZ, Braunschweig, Germany) were cultured in RPMI 1640 supplemented with 10% FCS, 100 µg/ml streptomycin and 100U/ml penicillin in saturated humidity at 37°C, 5%CO2. HUVECs were isolated from umbilical cords (kindly provided by D. Bernhard, Innsbruck, Austria [22]) and cultured in supplemented EGM2 (Lonza, Basel, Switzerland).

### Generation of Retro−/lentiviral Particles and Infection of Cells

Retro/lentiviral infection of target cells was performed as described previously [23]. In brief, for lentiviral infection, 10∧6 HEK293T cells were transfected with 2 µg pGLTR vectors, 1 µg pSPAX2 packaging and 1 µg pMD-G VSV-G-pseudotyping plasmids (both vectors were kindly provided by D. Trono, EPFL, Lausanne, Switzerland) using Metafectene (Biontex, Martinsried, Germany). Similarly, for retroviral infection, PHOENIX™ packaging cells were transfected with 3 µg pLib-TetR-KRAB-IRES-BlasS together with 1 µg pMD-G. Target cells were infected using 0.45 µm filtered virus containing cell culture supernatant obtained at 48 and 72 hours after transfection and supplemented with 4 µg/ml polybrene. 48h after infection cells were selected for puromycin- (1 µg/ml) or Blasticidin S resistance (5 µg/ml). U2OS cell lines expressing TetR (U2OS-TetR) were generated by lentiviral transduction using pLENTI6/TR (Invitrogen, Vienna, Austria) and selected for Blasticidin S resistance. Conditional RNAi in pGLTR superinfected cells was induced by addition of up to 1 µg/ml doxycycline for up to 72 hours. Generation of retro- and lentiviral particles and target cell infection were performed under biological safety 2 conditions.

### Immunoblotting

Total cell lysates were prepared by lysing 10∧6 cells in 100 µl SDS-sample buffer containing 5% 2β-mercaptoethanol. After boiling, 10 µl were separated by denaturing gel electrophoresis transferred to nitrocellulose membrane using semi-dry electroblotting and analysed by using antibodies against CDC27 (BD Transduction Lab, Vienna, Austria), α-tubulin (Tat-1, J. Gannon, Cancer Research UK, South Mimms, UK), BIM (BD Bioscience, Vienna, Austria), or GAPDH (mAb6C5, HyTest. Ltd., Turku, Finland) followed by enhanced chemiluminescence using HRP-conjugated secondary antibodies.

### Microscopy and Flow Cytometry

Life cell images were taken on inverse Zeiss Axiovert 200M (Carl Zeiss, Vienna, Austria) or Olympus IX70 (Olympus, Vienna, Austria) microscopes using 20x and 40x objectives. For sorting, 10∧6 PREB697/EU3 cells were infected simultaneously with lentiviral vectors expressing shRNA targeting Bim [23] and eGFP or dtTOMATO marker genes. Ninety six hours after infection, cells were analysed for their fluorescence intensities and sorted on a FACSVantage SE (BD, Vienna, Austria) equipped with 360nm, 488 nm and 633nm laser lines into single or double positive cells into separate vials. After sorting, 10∧6 cells were analysed for target gene expression by immunoblotting.

## Results

### TetO Flanked H1 Promoter THT for Conditional RNAi

**Figure 1:**
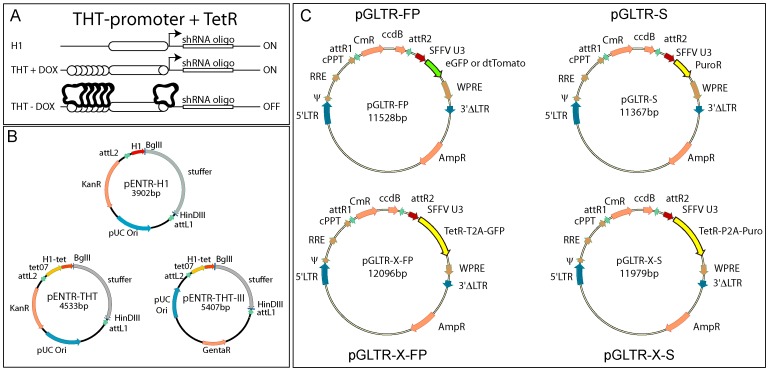
Overview of the GATEWAY-compatible lentiviral tetracycline-regulated RNAi (GLTR) RNAi system A). The H1-RNA core promoter constitutively drives the expression of a downstream shRNA encoding gene. TetO sequences (circles) allow the tetra−/doxycycline-dependent binding of TetR or TetR fusion proteins such as TetR-KRAB. B) GATEWAY compatible ENTR-plasmids for constitutive (pENTR-H1) and conditional (pENTR-THT and -THT-III) expression of shRNAs. C) GATEWAY compatible lentiviral destination (DEST) vectors. The fluorescence protein (FP) vectors encode GFP or RFP, the selectable vectors (S) puromycin or neomycin resistance. The one-vector system pGLTR-X encodes sequences for the simultaneous expression of GFP or puromycin resistance and the TetR-repressor. Abbreviations: H1, RNA-polymerase III promoter; THT, TetO sequences flanked H1 promoter; att (L and R) attachment site; KanR, kanamycin resistance; GentaR, gentamycin resistance; LTR, long terminal repeat; RRE, rev response element; cPPT, central polypurine tract; CmR, chloramphenicol resistance; ccdB, ccdB protein (inhibitor of E.coli DNA gyrase); SFFV U3, spleen focus forming virus U3 promoter; WPRE, woodchuck hepatitis posttranscriptional response element; AmpR, ampicillin resistance; T2A, 2A-like cis-acting hydrolase elements of Thosea asigna virus; P2A, 2A-like cis-acting hydrolase elements of porcine teschovirus.

To develop a conditional RNAi system, we constructed the THT-promoter by flanking the human H1-RNA gene promoter with Tet operator sequences, i.e., binding sites for bacterial TetR. First, a heptamerised Tet-operator was introduced upstream of the H1-promoter, which was then further modified by substituting the spacer between the TATA box and the transcription start site with a single TetR binding site (Fig. 1A). These modifications did not affect the activity of the H1-promoter but rendered it repressible in the presence of TetR or TetR-KRAB, which is a fusion protein of bacterial TetR with the transcription silencing KRAB domain of KOX1 [20] (data not shown).

The unmodified H1- as well as the THT-promoters were then subcloned into GATEWAY ENTR vectors to obtain pENTR-H1 (kanamycin resistance), pENTR-THT (kanamycin) as well as pENTR-THT-III (gentamycin) as shown in Figure 1B. Upon transient transfection of these plasmids into target cells, shRNA expression from these vectors is constitutive, which allows the rapid evaluation of the specificity and efficacy of the selected shRNA sequence to knock down target gene expression. Upon transfection into TetR or TetR-KRAB expressing cells, however, the THT promoter is repressed unless doxycycline is added to the media. Because the THT-shRNA expression cassette is flanked by attL1 and attL2 sequences, it can be readily transferred to various GATEWAY compatible vectors for efficient delivery into target cells.

### Lentiviral Fluorescent Protein Co-expression GLRT-FP Vectors (Two Vector System)

To evaluate the conditional RNAi system, we chose to target CDC27 (APC3), a subunit of the essential mitotic ubiquitin ligase anaphase promoting complex/cyclosome (APC/C) [24]. Loss of APC/C function prevents the degradation of mitotic cyclins and arrests cells in mitosis [25]. Thus, any leaky shRNA expression system would prevent the establishment of stable cell lines, while poor inducibility would fail to arrest cells in mitosis. The CDC27 targeting pENTR-THT construct was generated by subcloning a 64bp double-stranded oligonucleotide into the BglII-HinDIII sites of pENTR-THT. After sequence confirmation, expression of the shRNA by transient transfection into HeLa and U2OS cells was found to be effective in knocking down CDC27 levels (not shown).

Because RNAi experiments can require different gene delivery or expression strategies, we constructed three different types of vectors for **G**ATEWAY-compatible **l**entiviral **t**etracycline-regulated **R**NAi (**GLTR**, Fig. 1C). The first type of vectors was designed to allow the use of **f**luorescent **p**roteins (**FP**) (pGLTR-**FP**) for tracking transduced cells, for colour coded combinatorial RNAi and to have a proxy for RNAi induction in cells expressing TetR-KRAB. The second type of vectors was created to allow **s**election of transduced cells (pGLTR-**S**), while the third system was designed as a one vector approach for GFP labelling or puromycin resistance and conditional RNAi (pGLTR-X-FP and pGLTR-X-S, respectively).

To test the functionality of our two-vector system (pGLTR-FP and pHR-SFFV-TetR-KRAB-IRES-PURO), we used GATEWAY-recombination to generate pGLTR-FP-GFP-CDC27, in which GFP expression is controlled by the constitutive SFFV-promoter and CDC27 shRNA by the conditional THT promoter. Since TetR-KRAB is known to silence genes within up to 10 kb of its binding site [20], we expected not only conditional RNAi but also co-regulation of GFP in cell lines expressing TetR-KRAB.

**Figure 2:**
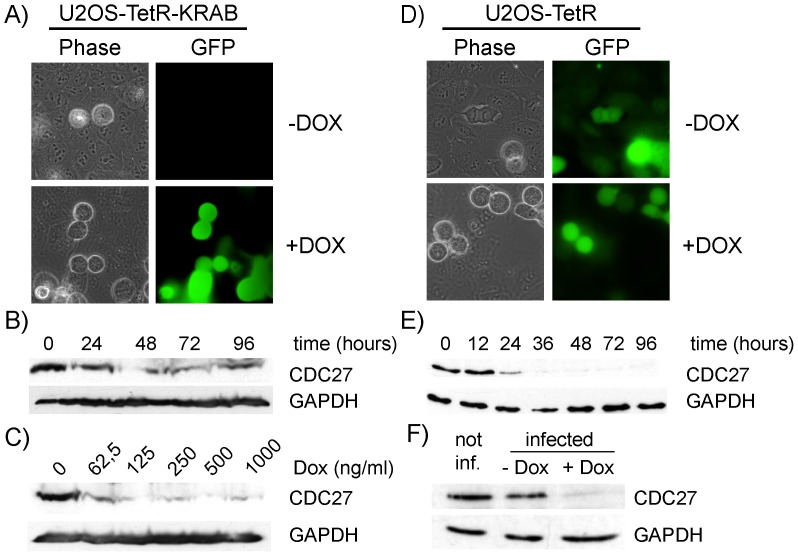
Conditional silencing using TetR-KRAB or TetR repressor proteins. U2OS cells constitutively expressing TetR-KRAB (A,B,C) or TetR (D,E,F) were infected with pGLTR-FP-GFP-CDC27 and cultured in the absence or presence of 1 µg/ml doxycycline (Dox) for 3 days (A,D). In the presence of TetR-KRAB, GFP expression was Dox dependent (A). For time-course experiments (B,E) cells were treated with 1 µg/ml Dox and samples taken at the indicated time points for immunoblotting for CDC27 levels as well as GAPDH as loading control. For dose-dependent experiments (C), cells were treated with increasing doses of Dox for 3 days and total cell extracts analysed for CDC27 and GAPDH expression. F) Inducibility and leakiness of TetR-regulated shRNA expression was tested in pGLTR-FP-GFP-CDC27 infected U2OS-TetR cells analysed for CDC27 and GAPDH expression. Shown are representative results from 3 independent experiments.

To test this prediction, we first generated U2OS cells constitutively expressing TetR-KRAB and then transduced these cells with lentiviral pGLTR-FP-GFP-CDC27 particles. As can be seen in Figure 2A, GFP was only expressed in transduced cells upon addition of doxycycline. A reduction in CDC27 levels became already detectable by immunoblotting 24 hours after induction by as little as 62.5 ng/ml doxycycline (Fig. 2B, C). In addition, knockdown of CDC27 caused an increase in rounded up cells (Fig. 2A) with chromosomes in a metaphase-like configuration (not shown), which is characteristic for the mitotic arrest caused by inhibition of the APC/C. These experiments showed that the THT promoter was effectively silenced by TetR-KRAB. Marker genes, such as GFP, expressed from the same vector, were also co-regulated by TetR-KRAB and could be used to monitor shRNA expression upon doxycycline treatment. GFP expression can, therefore, be used as a proxy for RNAi induction which might be useful in live cell imaging experiments allowing individual infected cells to be identified, traced and analysed according to their GFP fluorescence intensity. This system has already been used successfully for several other genes in various cell types by us and others [23], [26]. A drawback of TetR-KRAB is that it precludes the use of selection markers to enrich for transduced cells.

### Selectable Lentiviral GLTR-S Vectors (Two Vector System)

The efficacy of RNAi is dose-dependent and efficient lentiviral expression of shRNAs may, therefore, require multiple viral integrations, which can be achieved by high lentiviral titres or multiple rounds of infection. In both cases, selection of transduced cells might be required to establish efficient RNAi clones. To establish conditional and selectable RNAi, we investigated the use of TetR as a specific regulator of THT-promoter dependent shRNA gene expression that would not affect the expression of adjacent genes.

First, we generated U2OS cells constitutively expressing TetR by lentiviral infection and selection for Blasticidin S resistance. Resistant U2OS-TetR cells were then superinfected with pGLTR-FP-GFP-CDC27 and cultured in the presence of increasing amounts of doxycycline. In these cells, GFP expression was constitutive (Fig. 2D), while CDC27 knockdown was inducible in a time-dependent manner (Fig. 2E) similar to that observed in the above described TetR-KRAB-based system. To allow selection of transduced cells, we next exchanged the eGFP expression cassette with one encoding for puromycin resistance (pGLTR-S-PURO-CDC27) and infected U2OS-TetR cells with lentiviral GLTR-S-PURO-CDC27 particles. After puromycin selection, we compared CDC27 levels of uninfected and pGLTR-S-PURO-CDC27 transduced U2OS-TetR cells, cultured in the absence or presence of 1 µg/ml doxycycline for 72h (Fig. 2F). These experiments revealed that TetR was sufficient to repress transcription from the THT promoter in the absence of doxycycline, which allowed selection or enrichment strategies for transduced cells to rapidly establish stable RNAi cell lines.

### GLTR-FP Vectors for FACS

**Figure 3:**
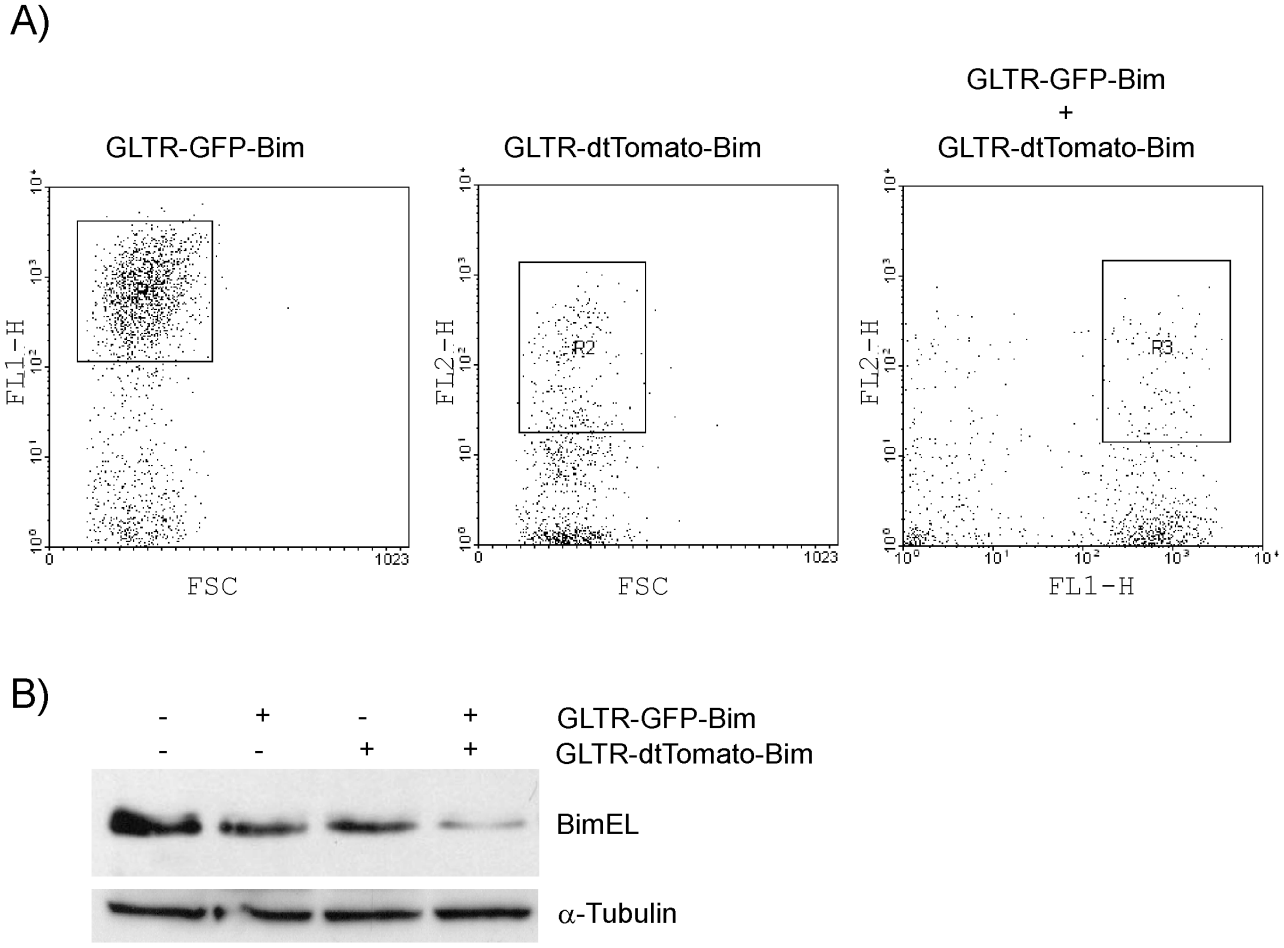
Fluorescent marker proteins enabling combinatorial RNAi. PreB697/EU3 cells were infected with lentiviral particles encoding a shRNA targeting BIM in combination with either eGFP- or dtTOMATO- fluorescence reporters. (A) PreB697/EU3 cells were sorted either for eGFP (left panel, R1), dtTOMATO- (middle panel, R2) or eGFP- and dtTOMATO- (right panel, R3) expression. (B) Protein lysates of sorted cells were analysed for BIM and α-tubulin expression by immunoblotting.

To demonstrate that the above described pGLTR-FP vectors can be used for flow cytometry-based enrichment, we chose the hard to transfect preB leukemic cell line PREB697/EU3. Since one round of infection is often insufficient for efficient gene knockdown in this cell line (unpublished observations), we generated the lentiviral vector pGLTR-FP-RFP by exchanging the GFP marker gene of pGLTR-GFP with the gene for red fluorescent dtTOMATO [27]. The fluorescence spectra of eGFP and dtTOMATO are well separated from each other and suitable for two colour fluorescence imaging and cell sorting (Fig. 3).

To evaluate dual colour-coded RNAi, we infected PREB697/EU3 cells with lentiviral RNAi vectors that target the pro-apoptotic BH3-only protein BIM [23] and co-expressed eGFP (pGLTR-GFP-BIM) or dtTOMATO (pGLTR-RFP-BIM). Infected cells were then sorted according to their fluorescence signals (Fig. 3A) and analysed for target gene knockdown by immunoblotting. As shown in Figure 3, cells that exhibited high fluorescence for both colours showed effective knockdown of BIM, while single coloured PREB697/EU3 cells exhibited less efficient knockdown (Fig. 3B). Thus, different labelling of the lentiviruses allows the simultaneous use of two or more pGLTR-FP or –S vectors for combinatorial RNAi experiments.

### Inducible and Selectable Lentiviral One-vector system: GLTR-X

The above described conditional RNAi systems are based on two components, the THT-shRNA expression cassette and a tetracycline-dependent repressor, each encoded by separate viral vectors (two-vector system). To overcome the need for sequential or co-infection of target cells, we designed pGLTR-X, which contains a GATEWAY-DEST cassette for uptake of the THT-shRNA gene and an expression cassette for a TetR variant with a C-terminal nuclear localisation signal (NLS) followed by a T2A sequence fused to eGFP driven by the constitutively active SFFV promoter. During translation, the T2A sequence induces ribosomal ‘skipping’ that causes stop codon independent peptide release and re-initiation of translation at the T2A site [28], [29] resulting in ‘cleavage’ of the fusion protein to produce TetR-NLS and eGFP.

**Figure 4:**
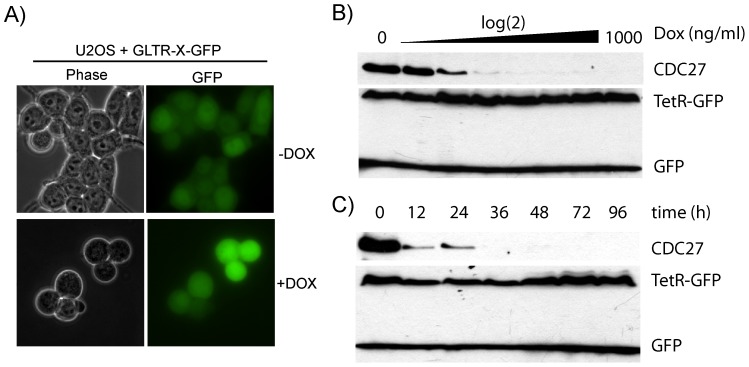
Conditional silencing using the one-vector GLTR-X system. U2OS cells were infected with pGLTR-X-GFP-CDC27. Infected cells were cultured in the presence or absence of 1 µg/ml Dox for 3 days and analysed by microscopy. Induced cells reveal a mitotic arrest phenotype (A). For time-course experiments (B), cells were treated with 1 µg/ml Dox and samples taken at the indicated time points for immunoblotting for CDC27 levels as well as GFP as loading control. For dose-response analysis (C), cells were treated with increasing doses of Dox for 3 days and total cell extracts analysed for CDC27 and GFP expression.

To examine whether our single vector system was sufficient for the generation of conditional RNAi cell lines, we transduced U2OS cells with pGLTR-X-GFP-CDC27 and analysed CDC27 levels upon doxycycline treatment. Similar to the two vector system, CDC27 levels were efficiently reduced in a time- and dose-dependent manner (Fig. 4B, C). As expected, induced pGLTR-X-GFP-CDC27-infected cells arrested in metaphase of mitosis (Fig. 4A). Consistent with previous findings [30], the T2A sequence between TetR-NLS and GFP resulted in about 50% of fusion protein ‘cleavage’ (Fig. 4B, C)[30].

**Figure 5:**
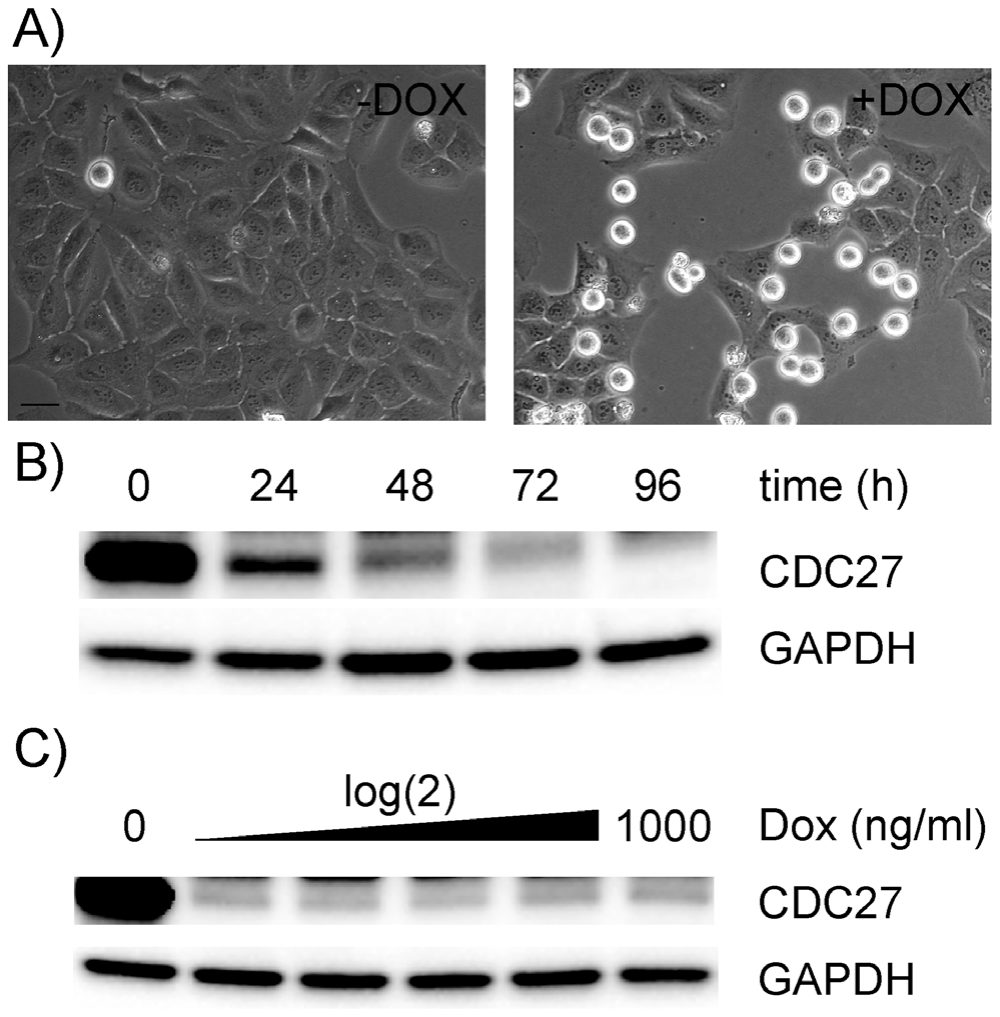
Conditional silencing using the selectable one-vector GLTR-X system. U2OS cells were infected with pGLTR-X-Puro-CDC27. Forty eight hours after infection cells were selected for puromycin resistance (1 µg/ml). Resistant cells were cultured in the presence or absence of 1 µg/ml Dox for 3 days and analysed by phase contrast microscopy (A). For time-course experiments (B), cells were treated with 1 µg/ml Dox and samples taken at the indicated time points for immunoblotting for CDC27 levels as well as GAPDH as loading control. For dose-response analysis (C), cells were treated with increasing doses of Dox for 3 days and total cell extracts analysed for CDC27 and GAPDH expression.

**Figure 6:**
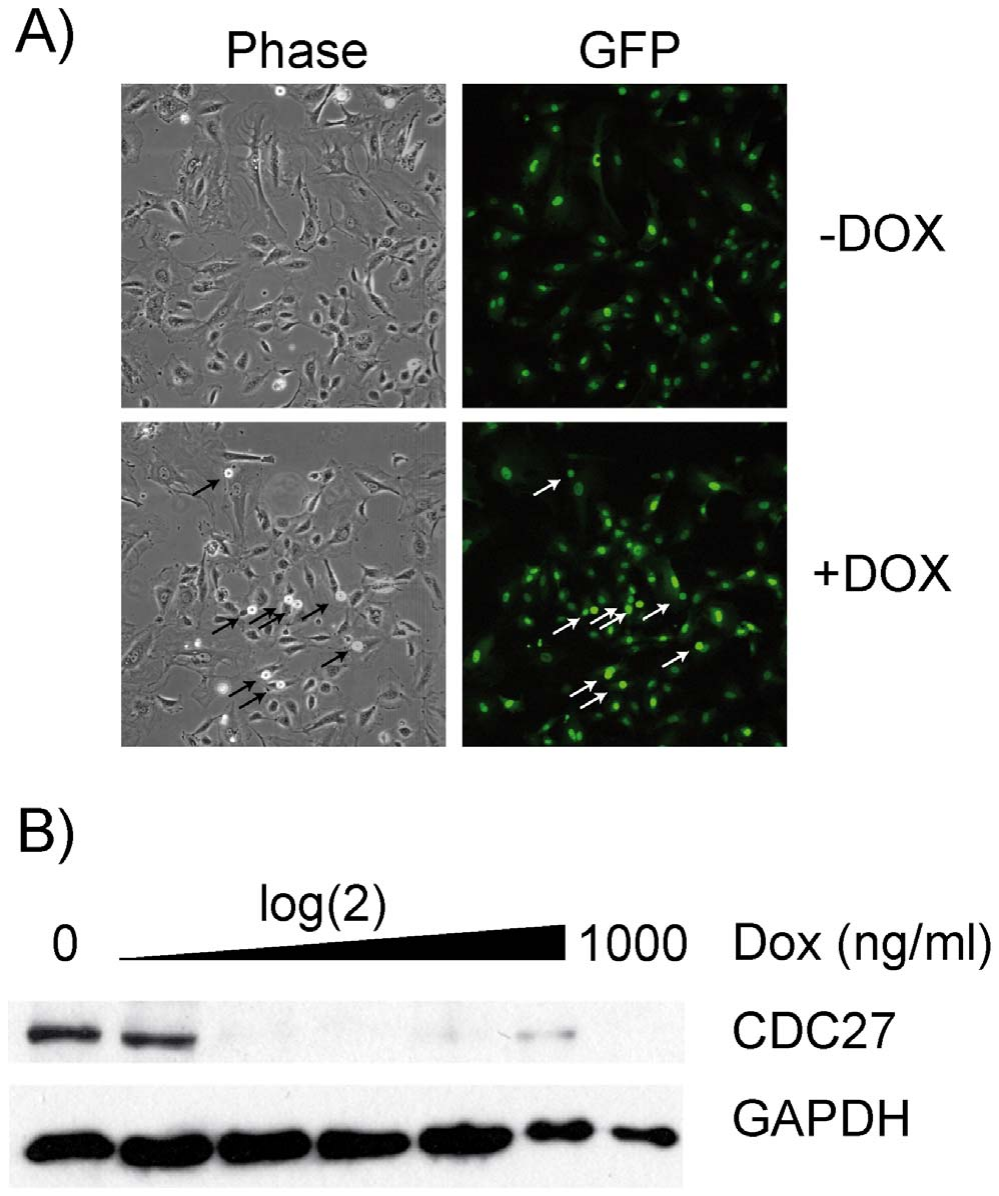
Conditional gene silencing in primary human HUVEC cells. pGLTR-X-GFP-CDC27 was used to infect primary HUVECs. Infected cells were induced 5 days after infection for 3 days with 1 µg/ml Dox and analysed by phase contrast and fluorescence microscopy. Mitotic cells are labelled by arrows (A). For dose-response analysis (B), cells were treated with increasing doses of Dox for 3 days and total cell extracts analysed for CDC27 and GAPDH expression.

Next, we exchanged the GFP cassette with a puromycin resistance sequence to enable selection and enrichment of infected cells. Infection of U2OS cells with pGLTR-X-PURO-CDC27 and induction with doxycycline resulted in mitotic arrest (Fig. 5A) and CDC27 knockdown in a time and dose-dependent manner (Fig. 5B, C). To test whether the single vector system, pGLTR-X, might be suitable for generating conditional RNAi in primary cells, we transduced HUVEC cells with lentiviral pGLTR-X-GFP-CDC27 particles. Treatment of infected cells with doxycycline resulted in mitotic arrest (Fig. 6A, arrows) and efficient CDC27 protein knockdown (Fig. 6B). Thus, a single infection of pGLTR-X is sufficient to enable conditional RNAi in primary cells.

## Discussion

The success of RNAi experiments critically depends on the expression levels of shRNAs or siRNAs, respectively. Too low expression levels may result in difficult-to-interpret hypomorphic phenotypes, while too high levels can interfere with the processing of endogenous small non coding RNAs, such as miRNAs [31], and increase the chance of off-target effects. Off-target effects are caused by sufficient similarities between the siRNA sequences (of both the sense and antisense strand) and cellular mRNAs other than the target molecule [31], [32]. By overloading the RISC or the RNA processing enzymes DROSHA and DICER, excessive shRNA expression levels might also interfere with the miRNA pathway with unpredictable and pleiotropic effects on cellular protein levels [33]. Both effects are dose-dependent and require careful titration of shRNAs/siRNAs for optimal knockdown efficiency and specificity.

To satisfy these requirements, we generated a novel modular RNAi system for stable and conditional RNAi. This system uses GATEWAY recombination-mediated transfer of a conditional promoter for shRNA expression into a set of novel lentiviral delivery vectors that can be used for establishing stable RNAi cell lines, combinatorial RNAi as well as conditional RNAi. To achieve conditional RNAi, we generated an H1-RNA gene derived promoter, THT, which enables conditional expression of transduced shRNAs in target cells expressing tetracycline-sensitive repressors, such as TetR-KRAB or TetR. The comparison of TetR-KRAB and TetR regulated shRNA expression suggested that both molecules are equally efficient to tightly control the induction of RNAi by repressing the activity of the THT promoter. Because TetR-KRAB induces silencing by recruiting HDACs to the THT promoter it can be used to monitor the induction of RNAi in cells transduced with lentiviral pGLTR-FP vectors. However, the use of TetR-KRAB has to be considered carefully because lentiviral integration is random and TetR-KRAB might also silence genes near the viral integration site [20].

Due to the spreading silencing effect of the KRAB domain, a selection gene to enrich for transduced cells can also not be used together with TetR-KRAB. In contrast, TetR, which acts by steric hindrance of RNA-polymerase-III-dependent shRNA transcription, effectively represses the THT promoter and allows the selection or enrichment of transduced cells if used in combination with pGLTR-S or pGLTR-FP vectors, respectively. Of course, all pGLTR-FP and pGLTR-S vectors can be used for constitutive, long-term RNAi experiments. The generation of pGLTR-FP vectors encoding different FACS-compatible fluorescence marker (GFP, dtTOMATO) further increases their applicability in hard to subclone or transfect cells or to enable combinatorial RNAi.

To facilitate the establishment of conditional RNAi cell lines in primary cells, which have a limited capacity to proliferate in vitro, we established a one vector system, which combines GFP labelling or puromycin resistance with TetR-based conditional RNAi. This approach enabled the generation of conditional RNAi in primary HUVEC cells after a single round of infection using pGLTR-X encoded viral particles. Since lentiviral particles can transduce non-proliferating cells, this system might be useful for any kind of conditional RNAi experiments in primary cells.
